# HPV vaccine hesitancy among Chinese adults and its influencing factors in the context of improved vaccine accessibility

**DOI:** 10.3389/fmed.2026.1838355

**Published:** 2026-05-25

**Authors:** Yanqiu Chen, Shiqi Xie, Jianrong Zhou, Hongjin Zhu

**Affiliations:** 1 Women and Children’s Hospital of Chongqing Medical University, Chongqing, China; 2 School of Nursing, Chongqing Medical University, Chongqing, China

**Keywords:** 3C model, HPV knowledge, HPV vaccine, influencing factors, vaccine hesitancy

## Abstract

**Background:**

HPV vaccination is crucial for preventing HPV-related diseases; however, vaccine hesitancy remains a global challenge. In China, given the increasing vaccine supply and optimized vaccination policies, understanding the current status and determinants of HPV vaccine hesitancy among adults is essential for improving vaccination coverage.

**Methods:**

A cross-sectional survey was conducted in China in 2024. Data were collected using a sociodemographic information form, an HPV knowledge questionnaire, the Vaccine Hesitancy Scale, and the WHO Vaccine Hesitancy 3Cs model. Descriptive statistics, chi-square tests, Mann–Whitney U tests, and binary logistic regression were utilized for data analysis.

**Results:**

A total of 967 adults participated in this study. The mean age was 33.41 years (SD = 8.71), and 84.4% were women. The overall HPV vaccine hesitancy rate was 19.86%, with 26.5% in men and 18.6% in women. Binary logistic regression analysis revealed that higher complacency, lower confidence and lower HPV knowledge were significant predictors of vaccine hesitancy.

**Conclusion:**

HPV vaccine hesitancy among adults is influenced by multiple factors, with higher complacency, lower confidence, and lower HPV knowledge being core issues. Future public health practices should develop personalized intervention strategies focused on improving HPV knowledge, particularly among men and older women. Concurrent efforts should be made to alleviate public complacency and rebuild vaccine confidence by disseminating scientific evidence through multiple channels to address cognitive and attitudinal barriers.

## 1 Introduction

Human papillomavirus (HPV) is among the most prevalent sexually transmitted pathogens globally. Nearly all sexually active individuals will be infected with HPV at some point in their lives (1). Persistent infection with high-risk HPV can lead to various cancers, including cervical, head and neck, and anogenital cancers. Based on 2018 data, HPV is estimated to have caused 620,000 cancer cases in women and 70,000 in men worldwide (2). In China, relevant studies predict that by 2030, the number of HPV-attributable cancer cases and the age-standardized incidence rate will reach 214,077 and 9.35 per 100,000, respectively (3).

The HPV vaccine is currently the most effective means of preventing HPV infections and associated cancers. Vaccination has been shown to provide effective clinical protection for at least 10 years (4). Moreover, the herd protection generated by HPV vaccination benefits unvaccinated women and men. For instance, a systematic review and meta-analysis demonstrated that HPV-related infections and diseases significantly decreased following the implementation of population-level vaccination programs (5). Another recent study indicated that, at the population level, HPV vaccination substantially reduces the risk of cervical cancer (6). Despite these benefits, a substantial gap remains between actual HPV vaccine coverage and the goal of achieving herd immunity. Data from the World Health Organization (WHO) shows that the average global HPV vaccine coverage rate is only 52% (7).

Over the past decade, China has gradually expanded its HPV vaccination policies. The bivalent HPV vaccine was approved in China in 2016 (8), followed by the 4-valent and 9-valent vaccines in 2017 and 2018, respectively. In 2019, the approval of a domestic HPV vaccine significantly improved accessibility and affordability for the Chinese population (9). By 2022, China expanded the eligible age range for the 9-valent vaccine to women aged 9–45 years (10). Additionally, although the HPV vaccine for men had not been officially approved during the period of this survey (2024), China formally approved the administration of the 4-valent HPV vaccine for men in January 2025 (11), indicating that the nationwide rollout of male HPV vaccination is underway. Despite these policy improvements and increased accessibility, vaccination rates among Chinese adults remain low. In 2022, the full-course HPV vaccination rate among women aged 9–45 in China was merely 6.21% (12). This discrepancy between low vaccination rates and improved vaccine accessibility may suggest the influence of vaccine hesitancy.

Vaccine hesitancy is defined as “a delay in acceptance or refusal of vaccination despite availability of vaccination services” (13) and has become a severe challenge to national immunization programs. Japan provides a notable example. In 2013, following extensive media reports of unconfirmed adverse events, Japan’s Ministry of Health, Labour, and Welfare withdrew its positive recommendation for the HPV vaccine. Consequently, the HPV vaccination rate dropped sharply from a peak of 70% to less than 1%, even though the vaccine remained within the national immunization program (14). In countries where the HPV vaccine is not included in immunization programs, hesitancy is also prominent. A systematic review and meta-analysis focusing on Chinese female college students and parents of girls indicated an HPV vaccine hesitancy rate of 31% (15). A growing body of research has examined factors influencing HPV vaccine hesitancy. For example, a review identified that socioeconomic status, gender, age, ethnicity, and religion significantly affect vaccine acceptance (16). Multiple studies have highlighted the association of social cognition, media influence, stigmatization, and misinformation with vaccine hesitancy (17, 18).

To systematically understand the complex causes of this phenomenon, the WHO proposed the “3C” model theoretical framework, comprising complacency, confidence, and convenience (13). Complacency refers to the underestimation of disease risk and doubts regarding the necessity of vaccination. Confidence pertains to trust in the effectiveness and safety of vaccines, as well as in the health system, professionals, and decision-makers. Convenience refers to vaccine accessibility, affordability, and social interaction effects. This model has been widely utilized to explore the drivers of vaccine hesitancy. For instance, a systematic review of empirical research in China noted that the primary reasons for vaccine hesitancy are low vaccine confidence, poor cognition, and inconvenience (15). A study in Serbia analyzed vaccine hesitancy among medical students based on the WHO 3C framework (19). A systematic review aimed at designing vaccine hesitancy interventions for adolescents and their parents used the 3C model to outline the barriers and facilitators of vaccination (20).

The Strategic Advisory Group of Experts on Immunization (SAGE) states that tackling vaccine hesitancy at a national or regional level requires a comprehensive understanding of its current prevalence, context, and determinants (21). Currently, most relevant studies focus on parents’ decisions regarding adolescent vaccinations, with relatively less attention paid to adults themselves. For adults, particularly older and married individuals, the potential benefit of vaccination may be perceived as uncertain due to the increased risk of prior HPV exposure, making their vaccination decisions more complex than those of adolescents. Therefore, against the backdrop of China expanding the eligible age for women’s HPV vaccination and the trend toward approving vaccination for men, understanding the decision-making mechanisms of this broader adult population is crucial for optimizing vaccination strategies. This study aims to explore the current status and underlying motives of vaccine hesitancy among adults, providing a basis for the design of precise interventions.

## 2 Materials and methods

### 2.1 Survey design and participant recruitment

A cross-sectional survey was conducted in China in 2024. Participants were recruited primarily from Chongqing, with some from other provinces. Convenience sampling and snowball sampling were employed. The inclusion criteria were: adults aged ≥18 years, Chinese citizenship, and the ability to comprehend and read Chinese. Participants were excluded if they did not complete the Vaccine Hesitancy Scale (VHS).

The sample size was calculated based on the HPV vaccine hesitancy rate observed in a pilot survey (16.18%). Using the formula $n=[{Z^{2}}_{\alpha /2}\times \pi \;(1-\pi )]/\delta^{2}$ , with α = 0.05, Z_α/2_ = 1.96, and δ = 0.03, the minimum required sample size was *n* = 579. Based on the 20% dropout rate, the sample size required for the study was at least 695.

During the initial phase of data collection, the VHS was administered only to unvaccinated participants using a skip-logic design. Following a protocol revision, the VHS was made mandatory for all participants. Consequently, the final analysis included only participants who completed the VHS (*n* = 967). The 254 participants who bypassed the VHS due to the initial skip logic were excluded from the analysis.

### 2.2 Survey procedure

The research team developed an online questionnaire using “Survey Star” platform (wjx.cn). In November 2023, a pilot survey was conducted to assess the feasibility of the survey instrument and to refine the questionnaire based on feedback. The formal survey was distributed via WeChat, the most widely used social platform in China. WeChat users were invited to participate and encouraged to share the survey link with their contacts. To ensure data quality, the following control measures were implemented: each WeChat account was restricted to a single submission to prevent duplicate responses, and questionnaires containing identical responses across all items were excluded from the analysis.

### 2.3 Survey instruments

Sociodemographic information collected included age, gender, educational level, occupation, marital status, monthly income per capita, and HPV infection history.

HPV vaccination decision-making autonomy was assessed using a single item: “Who makes the decision regarding your vaccination?.” Response options were: (1) Myself, (2) Spouse/partner, and (3) Joint decision.

Participants’ HPV knowledge was evaluated using five items: “Did you know about human papillomavirus (HPV),” “Did you know that HPV is mainly sexually transmitted?,” “Did you know that HPV is a significant contributor to cervical cancer?,” “Did you know that HPV can also cause genital warts and anal cancer in both men and women?,” and “Did you know about the HPV vaccine?.” Each item offered “Yes” or “No” options. Participants received one point for a “Yes” response and zero points for a “No” response.

The vaccine hesitancy scale (VHS), developed by the WHO’s Strategic Advisory Group of Experts (SAGE) on Immunization (22), has been widely applied in various countries and settings to assess hesitancy among both children and adults (23, 24). The scale comprises 10 items scored on a 5-point Likert scale (1 = strongly disagree, 2 = disagree, 3 = neither agree nor disagree, 4 = agree, 5 = strongly agree). Items 5, 9, and 10 are reverse-scored. The total score ranges from 10 to 50, with lower scores indicating higher vaccine hesitancy. In this study, a total score of ≤30 was defined as “hesitant,” representing a hesitancy level above the scale’s midpoint (25). The scale’s Cronbach’s *α* coefficient in this study was 0.776.

The WHO Vaccine Hesitancy 3Cs model (complacency, convenience, and confidence) (13) was utilized to explore the reasons underlying participants’ HPV vaccine hesitancy. Drawing on a prior study (26), 13 items were developed to operationalize the three dimensions: 3 items for complacency, 6 items for confidence, and 4 items for convenience. To evaluate construct validity, exploratory factor analysis (EFA) and reliability analysis were conducted for each subscale. For confidence, five items loaded strongly on this factor (loadings > 0.86), while one item about adverse event awareness was excluded due to negligible loading (−0.13) and low communality (0.017). The remaining five items Cronbach’s *α* = 0.933. For complacency, EFA supported a unidimensional structure (loadings ≥0.55). Cronbach’s α was 0.549; the mean inter-item correlation (MIIC) was 0.34, within the acceptable range for short scales (27). For convenience, EFA revealed two distinct factors: economic affordability (single price item; loading 0.99) and social influence/norms (three items; loadings ≥ 0.76; Cronbach’s *α* = 0.701). Convenience was split into two separate predictors accordingly. Detailed results are in Supplementary Tables S1, S2.

### 2.4 Statistical analyses

Data analysis was performed using IBM SPSS version 26.0 (Armonk, NY: IBM Corp). Categorical data were presented as frequencies and percentages, while continuous data were reported as means and standard deviations. Univariate analysis used chi-square tests for categorical variables, and Mann–Whitney U tests for continuous variables (HPV knowledge and 3C subscale scores) after Shapiro–Wilk tests confirmed non-normal distributions (all *p* < 0.05). Variables with a univariate *p* < 0.2, based on the recommendation of Hosmer and Sturdivant (28), were entered into a binary logistic regression model, with vaccine hesitancy (no hesitancy = 0, hesitancy = 1) as the dependent variable. Statistical significance was set at *p* < 0.05. To assess the robustness of the findings, two sensitivity analyses were conducted. First, a leave-one-out analysis was performed by excluding each HPV knowledge item one at a time and re-estimating the regression model. Second, the logistic regression was repeated using an alternative VHS cut-off of ≤ 40 to define vaccine hesitancy, following (25).

## 3 Results

### 3.1 Participants’ characteristics

A total of 967 participants were included in this study. Among them, 37.4% (362/967) were aged 31–40 years, 84.4% (816/967) were women, and 63.4% (613/967) were married. 51.7% (500/967) had an undergraduate degree or higher, 16.2% (157/967) were medical personnel, and 37.8% (366/967) reported a monthly per capita income of 3,000–5,999 yuan. 37.8% (365/967) reported a history of HPV infection or had never been screened, and 35.6% (344/967) reported that vaccination decisions were made jointly or solely by a spouse/partner. The participants’ characteristics are summarized in Table 1.

**Table 1:** Characteristics of participants (*N* = 967).

Variables	*N* (%)
Age	
18–24	152 (15.7)
25–30	260 (26.9)
31–40	362 (37.4)
41–45	76 (7.9)
>45	117 (12.1)
Gender	
Women	816 (84.4)
Men	151 (15.6)
Education level	
Junior college or below	467 (48.3)
Undergraduate	426 (44)
Postgraduate or above	74 (7.7)
Marital status	
Unmarried	293 (30.3)
Married	613 (63.4)
Other	61 (6.3)
Occupation	
Not employed	197 (20.4)
medical personnel	157 (16.2)
other occupations	613 (63.4)
Monthly income per capita	
<3,000	136 (14.1)
3,000–5,999	366 (37.8)
6,000–8,999	231 (23.9)
>9,000	234 (24.2)
HPV infection history	
Yes	197 (20.4)
No	602 (62.2)
Not screened	168 (17.4)
HPV vaccination decision-maker	
Myself	623 (64.4)
Joint decision	124 (12.8)
Spouse/Partner	220 (22.8)
Ever seen or heard adverse event reports of the HPV vaccine?	
Yes	444 (45.9)
No	523 (54.1)

### 3.2 HPV vaccine hesitancy

Overall, 19.86% (192/967) of participants reported vaccine hesitancy. The hesitancy rate was higher among men (26.5%, 40/151) than among women (18.6%, 152/816). By age group, hesitancy was 18% (74/412) among those aged 18–30, 20.4% (74/362) among those aged 31–40, and 22.8% (44/193) among those aged 41 and above. Participants with an undergraduate degree or higher had a hesitancy rate of 20.4% (102/500), compared to 19.3% (90/467) among those with a junior college or lower. Regarding HPV infection history, the hesitancy rate was 16.8% (33/197) among those with a history of HPV infection, 19.4% (117/602) among those without infection, and 25.0% (42/168) among those who had never been screened. With respect to vaccination decision-making, participants who made the decision themselves had a hesitancy rate of 18.6% (116/623); those whose spouse/partner decided had a rate of 15.4% (32/208), and those who made a joint decision had a rate of 32.4% (44/136). Further details are presented in Table 2.

**Table 2:** Univariate analysis of influencing factors of HPV vaccine hesitancy (*N* = 967).

Variables	HPV vaccine hesitancy		*χ*^2^/U	*p*
	Yes (*N* = 192)	No (*N* = 775)		
Participant characteristics				
Age			3.064	0.547
18–24	28 (14.6%)	124 (16.0%)		
25–30	46 (24.0%)	214 (27.6%)		
31–40	74 (38.5%)	288 (37.2%)		
41–45	20 (10.4%)	56 (7.2%)		
>45	24 (12.5%)	93 (12.0%)		
Gender			4.950	0.026
Women	152 (79.2%)	664 (85.7%)		
Men	40 (20.8%)	111 (14.3%)		
Education level			6.726	0.035
Junior college or below	90 (46.9%)	377 (48.6%)		
Undergraduate	95 (49.5%)	331 (42.7%)		
Postgraduate or above	7 (3.6%)	67 (8.7%)		
Marital status			1.488	0.475
Unmarried	53 (27.6%)	240 (31.0%)		
Married	124 (64.6%)	489 (63.1%)		
Other	15 (7.8%)	46 (5.9%)		
Occupation			2.285	0.319
Not employed	46 (24.0%)	151 (19.5%)		
Medical personnel	27 (14.1%)	130 (16.8%)		
Other occupations	119 (61.9%)	494 (63.7%)		
Monthly income per capita			4.330	0.228
<3,000	27 (14.1%)	109 (14.1%)		
3,000–5,999	61 (31.8%)	305 (39.4%)		
6,000–8,999	50 (26.0%)	181 (23.3%)		
>9,000	54 (28.1%)	180 (23.2%)		
HPV infection history			4.054	0.132
Yes	33 (17.2%)	164 (21.2%)		
No	117 (60.9%)	485 (62.6%)		
Not screened	42 (21.9%)	126 (16.2%)		
HPV vaccination decision-maker			3.360	0.186
Myself	116 (60.4%)	507 (65.4%)		
Joint decision	44 (22.9%)	92 (11.9%)		
Spouse/Partner	32 (16.7%)	176 (22.7%)		
Ever seen or heard adverse event reports of the HPV vaccine?			0.211	0.646
Yes	91 (20.5%)	353 (79.5%)		
No	101 (19.3%)	422 (80.7%)		
HPV Knowledge (Mean ± SD)	3.45 ± 1.69	4.13 ± 1.31	57766.5	<0.001
3C model (Mean ± SD)				
Complacency	6.68 ± 2.24	5.29 ± 1.92	47330.5	<0.001
Confidence	18.05 ± 4.13	20.15 ± 3.43	50,961	<0.001
Convenience				
Economic affordability	3.61 ± 0.95	3.69 ± 0.79	71,634	0.385
Social Influence/Norms	4.76 ± 1.21	5.15 ± 1.01	62,056	<0.001

^†^Continuous variables were non-normally distributed; Mann–Whitney *U* test was used for group comparisons.

### 3.3 Influencing factors of HPV vaccine hesitancy

Univariate analysis identified the following variables as significantly associated with vaccine hesitancy: gender (*χ*^2^ = 4.950, *p* = 0.026), education level (*χ*^2^ = 6.726, *p* = 0.035), HPV knowledge (*U* = 57766.5, *p* < 0.001), complacency (*U* = 47330.5, *p* < 0.001), confidence (*U* = 50,961, *p* < 0.001), and social influence/norms (*U* = 62,056, *p* < 0.001). Variables with *p* < 0.2 in the univariate analysis, including HPV infection history (*p* = 0.132) and HPV vaccination decision-maker (*p* = 0.186) were also entered into the binary logistic regression model. Details are shown in Table 2.

After adjusting for all covariates, confidence (OR = 0.91, 95% CI: 0.86–0.96, *p* = 0.001) and HPV knowledge (OR = 0.87, 95% CI: 0.75–1.00, *p* = 0.046) were significantly associated with lower odds of vaccine hesitancy, while complacency (OR = 1.20, 95% CI: 1.08–1.33, *p* = 0.001) was associated with higher odds. Participants with an undergraduate degree were also more likely to be hesitant compared to those with a junior college degree or below (OR = 1.44, 95% CI: 1.01–2.06, *p* = 0.044). Table 3 presents the results of the binary logistic regression.

**Table 3:** Binary logistic regression analysis of influencing factors of HPV vaccine hesitancy (*N* = 967).

Variables	*B*	SE	Wald *χ*^2^	*p*	OR	95%CI
Gender						
Men (reference)	−0.230	0.237	0.937	0.333	0.80	0.50–1.27
Women						
Education level						
Junior college or below (reference)			6.836	0.033		
Undergraduate	0.366	0.181	4.075	0.044	1.44	1.01–2.06
Postgraduate or above	−0.470	0.440	1.139	0.286	0.63	0.26–1.48
HPV infection history						
Yes (reference)			1.78	0.411		
No	−0.131	0.235	0.309	0.579	0.88	0.55–1.39
Not screened	0.166	0.296	0.315	0.575	1.18	0.66–2.11
HPV vaccination decision-maker						
Myself (reference)			1.863	0.394		
Spouse/Partner	−0.001	0.253	0.000	0.996	1.00	0.61–1.64
Joint decision	−0.287	0.218	1.731	0.188	0.75	0.49–1.15
HPV Knowledge	−0.144	0.072	3.964	0.046	0.87	0.75–1.00
3C model						
Complacency	0.180	0.054	11.184	0.001	1.20	1.08–1.33
Confidence	−0.091	0.028	10.793	0.001	0.91	0.86–0.96
Social influence/norms	0.017	0.095	0.031	0.861	1.02	0.84–1.23

### 3.4 Sensitivity analysis

A leave-one-out analysis for HPV knowledge confirmed that the two core predictors (confidence and complacency) remained significant across all models. HPV knowledge itself remained significant and showed stronger protective effects when the three items with the highest public awareness were excluded one at a time (ORs: 0.83–0.84, all *p* < 0.05). In contrast, when the two more basic items were removed, knowledge became non-significant (*p* > 0.10), suggesting that these items may have limited discriminatory power (Supplementary Table S3).

When vaccine hesitancy was defined using the ≤40 cut-off, the pattern of significant predictors remained largely unchanged. Complacency (OR = 1.27, 95% CI: 1.12–1.44, *p* < 0.001) and confidence (OR = 0.86, 95% CI: 0.81–0.92, *p* < 0.001) remained highly significant. Education level (*p* = 0.165) and HPV knowledge (*p* = 0.144) were no longer statistically significant, which is expected for variables with marginal *p*-values in the primary model. All other results were consistent with the primary model (Supplementary Table S4).

## 4 Discussion

This study examines the current status and influencing factors of HPV vaccine hesitancy among Chinese adults. The results indicate that 19.86% of adults reported hesitancy toward HPV vaccination, with complacency, confidence and HPV knowledge as the primary determinants.

The study found that approximately one-fifth of respondents reported HPV vaccine hesitancy. This rate is lower than those previously reported in studies concerning adolescents or their parents (29, 30). This discrepancy may be ascribed to differences in socio-demographic characteristics and policy contexts. In this research, the participant pool included both men and women across a broader age range. The expansion of the eligible vaccination age for women may have encouraged those previously ineligible to seek vaccination. Similarly, the emerging availability of male vaccination channels might have encouraged some men, particularly those with HPV knowledge, to get vaccinated. Moreover, new policies may have sparked heated discussions on social media, creating a “vaccination trend” that may have reduced hesitancy. Notably, even with policy promotion, this level of hesitancy persisted.

Vaccine hesitancy was higher in men than women (26.5% vs. 18.6%), which is consistent with previous studies (31, 32). Currently, HPV-related health education predominantly targets women, particularly in the context of cervical cancer prevention. Research exploring the impact of gender-biased information on health behaviors suggests that the female-oriented focus of HPV vaccination information, which frequently underscores feminine norms, limits the effectiveness of HPV health education for men (33). Moreover, societal attention regarding the risk of HPV infection in men is scarce, leading to complacency among them, as they may perceive themselves as unlikely to contract the virus (32, 34). Additionally, traditional gender norms may act as a deterrent, discouraging men from seeking help and accessing preventive healthcare services. A study analyzing public attitudes toward male HPV vaccination via social media data emphasizes that deep-rooted gender norms and biases hinder male HPV vaccination (35). Notably, in the logistic regression analysis, gender lost its statistical significance. This suggests that the higher vaccine hesitancy observed among men may be largely explained by differences in HPV knowledge, risk perception, and related cognitive factors, rather than being directly attributable to gender itself. Future studies employing formal mediation analysis are warranted to confirm this hypothesized pathway.

Consistent with previous studies (32, 36), HPV knowledge emerged as a crucial factor in reducing vaccine hesitancy. HPV-related knowledge not only enhances an individual’s risk perception but also increases trust in the necessity and safety of the vaccine. For example, a survey of female college students in China found that knowledge directly influences vaccination intentions and is mediated by trust and risk perception (36). A focus group of middle-aged adults also emphasized the need for more personalized information regarding age-specific safety and efficacy to facilitate informed decisions (37). This highlights the imperative of strengthening public health education. Notably, a leave-one-out sensitivity analysis revealed that the protective effect of knowledge was further strengthened when three items with the highest public awareness (e.g., “Did you know about the HPV vaccine”) were excluded, yet became non-significant when two more basic items were removed. This suggests that the original five-item scale may include items with limited discriminatory power, which may dilute the true protective effect of knowledge. Future studies should refine HPV knowledge scales by including items with greater variability to better capture individual differences in understanding. Interestingly, education level showed a divergent pattern. In this study, participants with an undergraduate degree exhibited higher hesitation than those with a junior college degree or below. This phenomenon, sometimes termed the “knowledge paradox,” has been observed in previous HPV research (38). This suggests that knowledge and education, though related, may influence vaccine hesitancy through distinct pathways, and interventions should not assume that higher education automatically translates to lower hesitancy.

Complacency and confidence were significant predictors of vaccine hesitancy, consistent with previous findings (39–41). Complacency reflects an individual’s underestimation of the risk of HPV infection. This cognitive bias is particularly pronounced in the adult population, possibly stemming from neglect of asymptomatic infection or lack of awareness of delayed consequences. Research (42) shows that many adults hold beliefs such as “HPV infection is not common” or “I won’t get cancer.” Gender role perceptions may exacerbate this phenomenon, with men often believing that HPV infection is “irrelevant to them” and women avoiding discussions due to cultural taboos (32, 43, 44). Confidence, meanwhile, reflects the impact of a vaccine confidence crisis. An analysis of HPV vaccine narratives on Twitter noted that concerns regarding vaccine safety, effectiveness, and mistrust in institutions were the primary drivers of vaccine hesitancy (45). Kutz et al. highlighted that concerns about potential side effects, such as impacts on fertility, may deter individuals from getting vaccinated (46). In the current era of information overload, the rapid spread of misleading information about the HPV vaccine further exacerbates hesitancy. Additionally, many healthcare workers exhibit vaccine hesitancy in both their personal and professional lives. A nationwide study of female healthcare professionals in Saudi Arabia found that more than half of the participants were unwilling to be vaccinated, with a lack of knowledge and specific misconceptions identified as the primary reasons for their hesitation (47). In the present study, although healthcare workers showed the lowest hesitancy rate (14.1%) compared to other occupations (19.2%) and the unemployed population (24.0%), the statistical analysis did not reveal a significant difference. This suggests that while a professional medical background may offer a certain protective effect, the current vaccine hesitancy crisis may have permeated groups across different occupational backgrounds. In China, healthcare workers are key trusted intermediaries in vaccine promotion; their hesitation may weaken public acceptance of new policies, especially during the early stages of vaccine rollout for expanded-age women and men.

In previous studies, convenience has often been identified as another key factor in HPV vaccine hesitancy (40, 41). However, in this study, the two dimensions of convenience, namely social influence/norms and economic affordability, showed limited predictive power. Social influence/norms were significant in univariate analysis but lost significance in the multivariable model, suggesting that their effect may be largely explained by vaccine confidence. Economic affordability was not significant in either the univariate or multivariate analysis. These findings suggest that adults’ vaccination decisions are driven more by internal cognitive and attitudinal factors than by external convenience. When complacency and confidence are prominent, the influence of convenience may be partly overshadowed. Therefore, intervention strategies that solely focus on improving vaccination convenience (e.g., social persuasion or cost reduction) may have limited effectiveness among adults. Addressing complacency and confidence, that is, tackling fundamental cognitive and attitudinal barriers, may be more critical. The finding that convenience was not an independent predictor, while complacency and confidence were, suggests a potential shift in the relative importance of barriers, moving from accessibility issues toward cognitive and attitudinal factors, in the current Chinese context. Further longitudinal research is needed to confirm this hypothesized transition.

### 4.1 Limitations and future directions

This study has several limitations. First, convenience and snowball sampling may lead to selection bias and limit the generalizability of the results. Second, the cross-sectional design restricts the ability to determine causal relationships between variables. Third, the HPV knowledge items were phrased as “Did you know…?”, which may induce acquiescence bias; however, a leave-one-out sensitivity analysis suggested that this wording did not undermine the validity of the findings, rather the analysis indicated that certain items had limited discriminatory power. Finally, the questionnaire did not thoroughly investigate the specific channels through which respondents obtained vaccine information, nor the detailed concerns underlying hesitation, which limits the ability to design tailored intervention strategies.

Future research could employ stratified random sampling and longitudinal tracking designs to enhance sample representativeness and causal inference. Furthermore, questionnaire content should be optimized to include HPV knowledge items with greater variability and a more detailed investigation of information sources and concerns, thereby providing more targeted guidance for interventions. Finally, empirical intervention programs integrating qualitative research findings could be designed to verify the effectiveness of strategies and promote public health practice.

## 5 Conclusion

This study found that the overall HPV vaccine hesitancy rate among Chinese adults was 19.86%, with 26.5% in men and 18.6% in women. Higher complacency, lower confidence, and lower HPV knowledge were identified as significant independent predictors of vaccine hesitancy. Future public health practices should prioritize the development of personalized intervention strategies, with a focus on enhancing HPV-related knowledge and awareness, particularly among men and older women. Concurrent efforts should be made to mitigate public complacency and rebuild vaccine confidence by disseminating scientific evidence and safety data through multiple channels. These measures aim to eliminate cognitive and attitudinal barriers, thereby increasing HPV vaccine uptake.

## Data Availability

The raw data supporting the conclusions of this article will be made available by the authors, without undue reservation.
