# Supplementary material for: HPV vaccine hesitancy among Chinese adults and its influencing factors in the context of improved vaccine accessibility
Source: Front Med (Lausanne). 2026 May 25;13:1838355. doi: 10.3389/fmed.2026.1838355 (PMC13243398; doi:10.3389/fmed.2026.1838355)
Supplement: Supplementary file 1 [file Table_1.DOCX]

**Supplementary Table S1**

| Dimension | | Item | Response Options (Score) | Cronbach’s α |
| --- | --- | --- | --- | --- |
| Complacency  (higher score = more complacency) | | Perceived prevalence of HPV-related cancer in China | High = 1; Medium = 2; Low = 3; Unclear = 4 | 0.549 |
|  |  | Perceived necessity of HPV vaccination | Very necessary = 1; Necessary = 2; Not certain = 3; Unnecessary = 4; Very unnecessary = 5 |  |
|  |  | Perceived vaccine effectiveness | Very effective = 1; Somewhat effective = 2  Neutral = 3; Not very effective = 4; Not effective at all = 5 |  |
| Confidence  (higher score = more confident) | | Trust in imported vaccine safety | Very distrustful = 1; Somewhat distrustful = 2; Neutral = 3; Somewhat trusting = 4; Very trusting = 5 | 0.933 |
|  |  | Trust in domestic vaccine safety |  |  |
|  |  | Trust in domestic manufacturers |  |  |
|  |  | Trust in foreign manufacturers |  |  |
|  |  | Trust in healthcare provider recommendations |  |  |
| Convenience  (higher score = greater convenience) | Economic affordability | Perceived price | Very low=5; Low = 4; Moderate = 3; High = 2; Very high=1 |  |
|  | Social influence/norms | Vaccination of close contacts | Yes = 2; No/Don’t know = 1 | 0.701 |
|  |  | Recommendation from friends/family |  |  |
|  |  | Recommendation from healthcare provider |  |  |

**Supplementary Table S2 Summary of the Reliability and Validity of the 3C Subscales**

| **Subscale** | **Items (n)** | **Factor Loading Range** | **Variance Explained (%)** | **Cronbach’s α** | **MIIC** |
| --- | --- | --- | --- | --- | --- |
| Complacency | 3 | 0.549-0.861 | 56.9 | 0.549 | 0.34 |
| Confidence | 5 (trust items) | 0.862-0.926 | 79.1 | 0.933 |  |
| Convenience (total) | 4 | - | 72.4 (two factors) | - | 0.20 |
| economic affordability | 1 (price) | 0.99 | - | - |  |
| social influence/norms | 3 | 0.76-0.85 | - | 0.701 | - |

Note: MIIC = mean inter-item correlation.

**Supplementary Table S3 HPV Knowledge Leave-One-Out Sensitivity Analysis**

| **Model** | **Excluded Knowledge Item** | **OR for Complacency (*p*)** | **OR for Confidence (*p*)** | **OR for HPV Knowledge (new score) (*p*)** |
| --- | --- | --- | --- | --- |
| 1 | Did you know about the HPV vaccine? | 1.20 (0.001) | 0.91 ( 0.001) | 0.83 (0.019) |
| 2 | Did you know that HPV is a significant contributor to cervical cancer? | 1.20 (0.001) | 0.91 (0.001) | 0.84 (0.020) |
| 3 | Did you know that HPV can also cause genital warts and anal cancer in both men and women? | 1.20 (0.001) | 0.91 (0.001) | 0.83 (0.036) |
| 4 | Did you know that HPV is mainly sexually transmitted? | 1.21 (0.001) | 0.92 (0.001) | 0.88 (0.174) |
| 5 | Did you know about human papillomavirus (HPV) | 1.21 (0.001) | 0.92 (0.001) | 0.89 (0.204) |

Note: OR = odds ratio. All models include the same covariates as the primary model. Core variables (confidence and complacency) remained significant in all models.

**Supplementary Table S4 Comparison of VHS Threshold Sensitivity Analysis**

| **Variables** | **Primary Model (≤30) OR (95% CI)** | ***P*** | **Sensitivity Model (≤40) OR (95%CI)** | ***P*** |
| --- | --- | --- | --- | --- |
| **Gender** |  |  |  |  |
| Men (reference) | 0.80 (0.50 - 1.27) | 0.333 | 0.82 (0.49 - 1.38) | 0.440 |
| Women |  |  |  |  |
| **Education level** |  |  |  |  |
| Junior college or lower (reference) |  | 0.033 |  | 0.331 |
| Undergraduate | 1.44 (1.01 - 2.06) | 0.044 | 1.27 (0.89 - 1.80) | 0.188 |
| Postgraduate or above | 0.63 (0.26 - 1.48) | 0.286 | 1.43 (0.73 - 2.78) | 0.294 |
| **HPV infection history** |  |  |  |  |
| Yes (reference) |  | 0.411 |  | 0.819 |
| No | 0.88 (0.55 -1.39) | 0.579 | 1.01 (0.67 -1.53) | 0.955 |
| Not screened | 1.18 (0.66 - 2.11) | 0.575 | 087 (0.50 - 1.52) | 0.624 |
| **HPV vaccination decision-maker** |  |  |  |  |
| Myself (reference) |  | 0.394 |  | 0.029 |
| Spouse/Partner | 1.00 (0.61 - 1.64) | 0.996 | 1.67 (0.88 - 3.15) | 0.115 |
| Joint decision | 0.75 (0.49 - 1.15) | 0.188 | 0.69 (0.45 - 1.04) | 0.074 |
| **HPV Knowledge** | 0.87 (0.75 - 1.00) | 0.046 | 0.88 (0.74 - 1.05) | 0.144 |
| **3C model** |  |  |  |  |
| Complacency | 1.20 (1.08 - 1.33) | 0.001 | 1.27 (1.12 - 1.44) | *P* < 0.001 |
| Confidence | 0.91 (0.86 - 0.96) | 0.001 | 0.86 (0.81 - 0.92) | *P* < 0.001 |
| Social influence /norms | 1.02 (0.84 - 1.23) | 0.861 | 0.93 (0.76 - 1.14) | 0.478 |

Note: All models include the same covariates. Only the VHS cut-off differs.
